# Case Report: Multiple primary bilateral lung benign notochord cell tumors combined with sacral tumors

**DOI:** 10.3389/fonc.2025.1537943

**Published:** 2025-05-21

**Authors:** Minjia Tao, Peijun Gao, Jiajie Liu, Biao Zhang, Guowei Tao

**Affiliations:** ^1^ Department of Thoracic Surgery, Haiyan People’s Hospital, Jiaxing, Zhejiang, China; ^2^ Department of Pathology, Haiyan People’s Hospital, Jiaxing, Zhejiang, China

**Keywords:** benign notochord cell tumor, pulmonary nodules, lung tumor, notochord tumor, surgery

## Abstract

Benign notochord cell tumor (BNCT) in the bilateral lungs is exceedingly rare. This report details a case of a male patient diagnosed with primary bilateral lung BNCTs and concurrent sacral tumors. As part of a routine health check-up, a chest CT revealed suspicious nodules in both lungs. We performed a thoracoscopic wedge resection of the nodules on him. The postoperative pathological findings combined with CT and clinical manifestations confirmed BNCT. Post-operatively, considering that BNCT commonly occurs in the axial skeleton, we conducted an examination of the spine. Spinal CT and MRI revealed suspicious sacral lesions in the S3 to S5 vertebrae, necessitating further investigation and continuous follow-up monitoring. The simultaneous occurrence of primary multifocal pulmonary BNCTs coexisting with sacral tumors has never been reported before. Fully taking into account potential associations with other diseases, careful follow-up and a comprehensive diagnostic approach are essential for patients with pulmonary nodules.

## 1 Introduction

In the 2020 WHO classification of bone tumors, BNCT is characterized as a spinal tumor that is typically asymptomatic and often discovered incidentally during imaging examinations of the head or spine (1). This article presents a case involving a male patient discovered to have suspicious pulmonary nodules on CT during a routine health check-up. Resection led to the diagnosis of BNCT. A postoperative examination identified a tumor focus in the sacral spine. To our knowledge, there are no existing reports of primary BNCTs involving both lungs along with sacral tumors.

## 2 Case description

**Figure 1:**
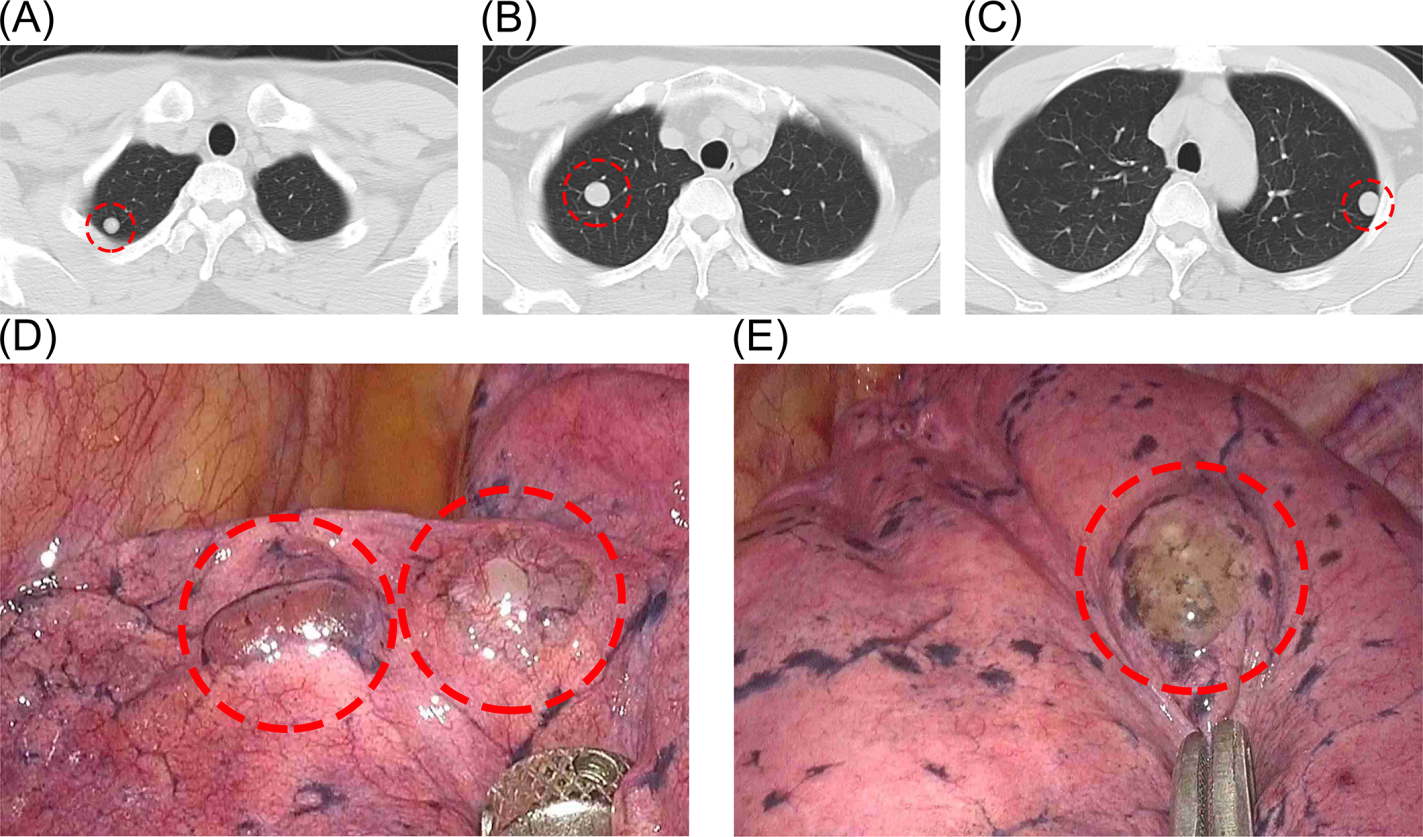
**(A–C)** Preoperative CT showing multiple solid nodules in both lungs. **(D, E)** Lesions visible under thoracoscopy.

The patient is a 44-year-old male, non-smoker, who has been working in the communications industry as a manager. He was found to have lung nodules and mediastinal mass during a routine health check-up one year ago. Over the past year, with regular follow-up exams, CT revealed multiple solid nodules in both lungs with well-defined boundaries (**Figures 1A–C**). The largest lung nodule measured approximately 16mm × 15mm. A distinct nodule approximately 16mm in diameter was also noted in the anterior superior mediastinum, characterized by smooth edges. The patient had no obvious clinical symptoms such as chest pain or hemoptysis. Considering the possibility of malignant transformation of the nodules in the future, the patient strongly requested surgical resection of the pulmonary nodules to obtain histological confirmation and clarify their benign or malignant nature. So we performed surgery on the patient. Before the operation, basic tests such as blood routine were performed to ensure that the patient could tolerate the operation. Thoracoscopic wedge resection of the lung nodules (**Figures 1D, E**) and mediastinal mass was performed, during which no pleural adhesions or effusions were observed. The excised tissues were sent for pathological examination and immunohistochemistry. The patient recovered well after the operation.

**Figure 2:**
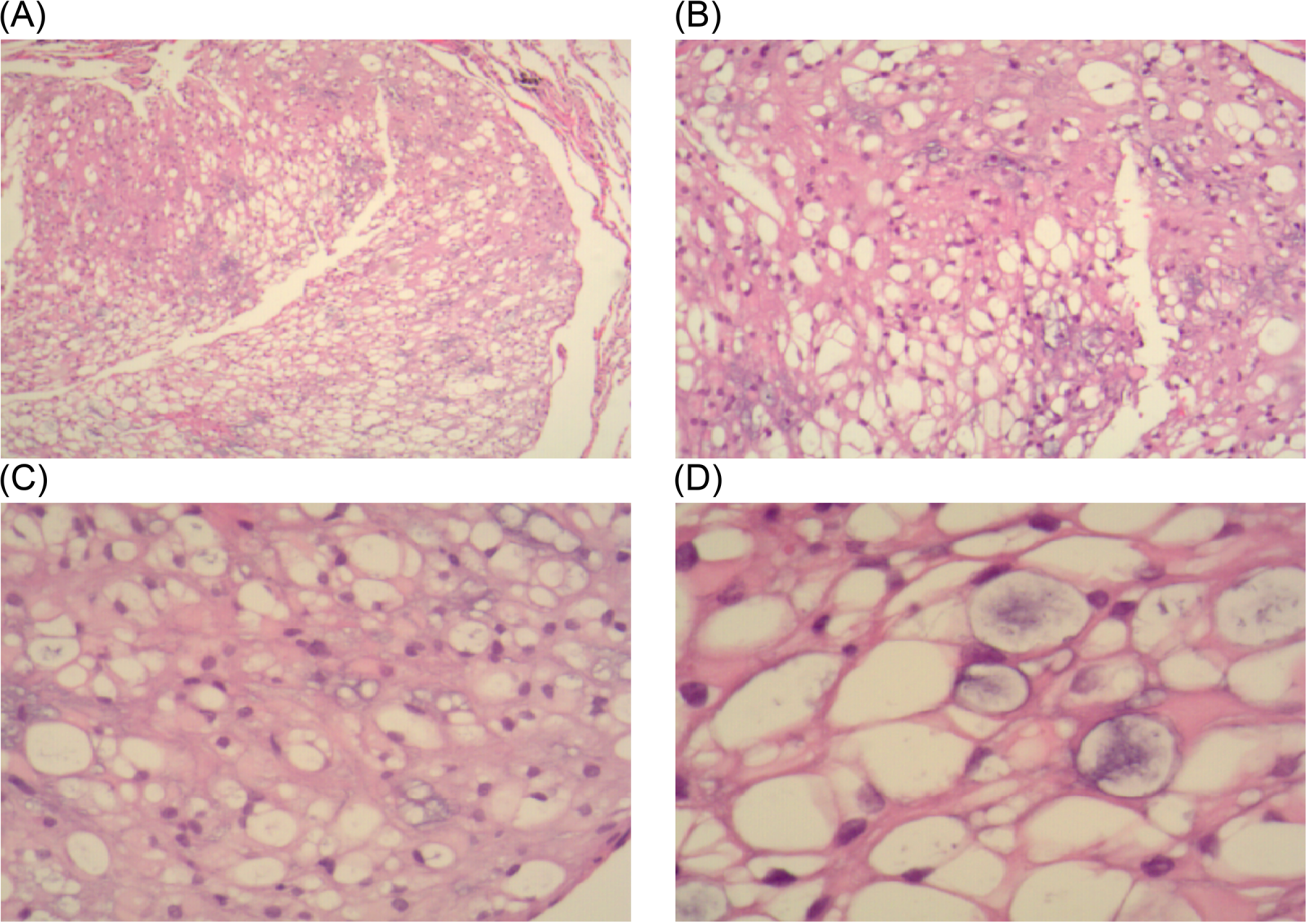
In the HE-stained pathological sections: **(A, B)** under 4x and 10x objectives lenses, tumor cells are diffusely distributed and have a loose structure. **(C, D)** under 20x and 40x objective lenses, a large number of vacuolated cells can be seen.

**Figure 3:**
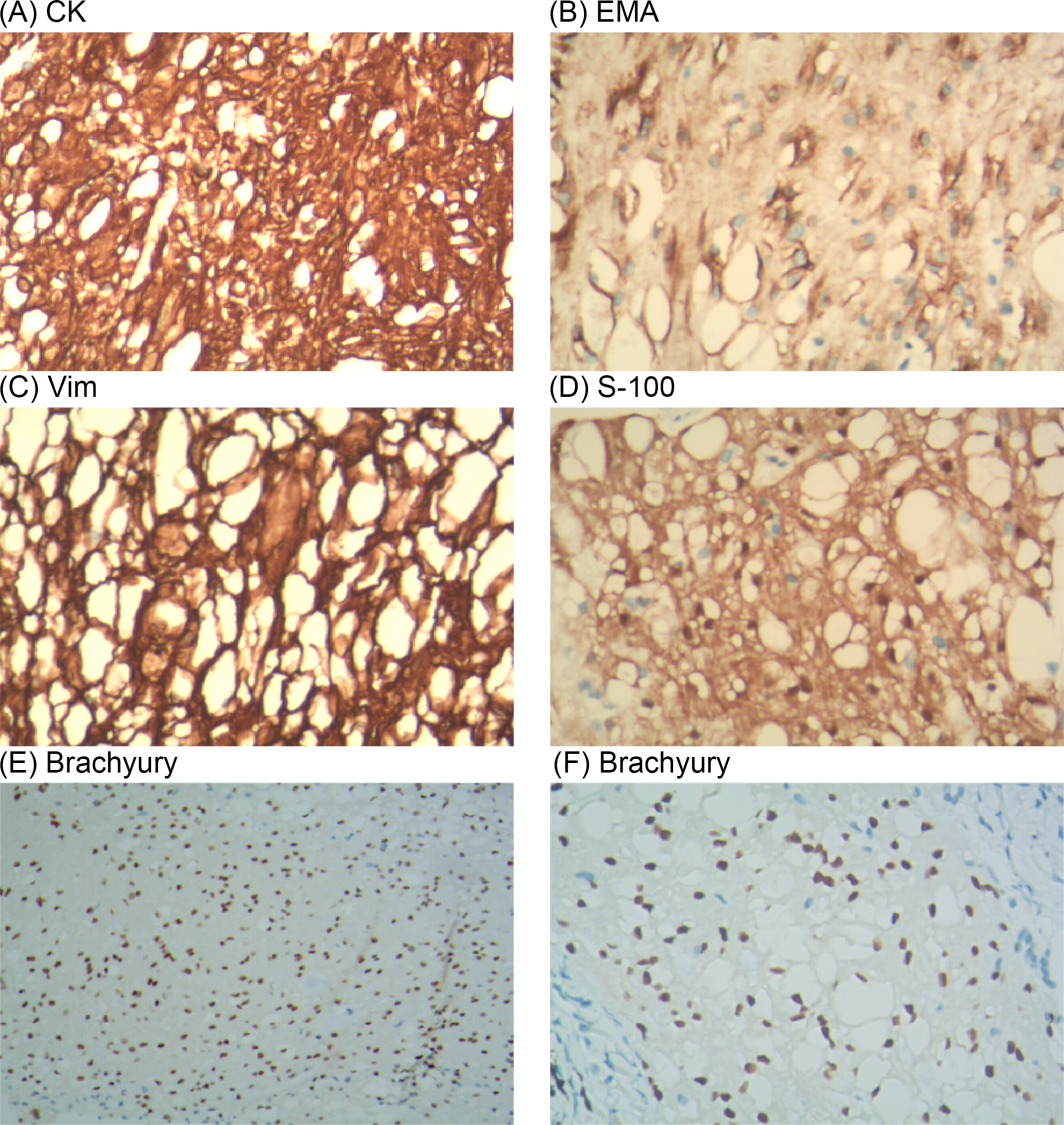
On immunohistochemistry analysis, the surgical specimen was positive for CK **(A)**, EMA **(B)**, Vim **(C)**, S-100 **(D)** and Brachyury **(E, F)**.

The mediastinal mass was identified as a tracheogenic cyst in the pathological report and was deemed normal. The nodular lung lesions exhibited lobulated growth and occasional nuclear divisions, with a significant presence of vacuolated cells observed (**Figure 2**). Immunohistochemistry results (**Figure 3A–D**): S-100 (+), CK (pan) (+), D2-40 (–), SMA (-), HMB45 (-), PR (-), EMA (+), Vim (+), Ki-67 (+, 5%), These findings led to a diagnosis of the notochordal cell tumor. To further confirm the notochordal origin of the tumor, we performed immunohistochemical testing of brachyury (transcription factor for notochord cell differentiation) on the pathological specimens again, and the results were positive (**Figures 3E, F**), which supported the diagnosis of notochordal origin of the tumor. Based on all collected data, the pulmonary nodules in this case were considered to represent multiple BNCTs.

**Figure 4:**
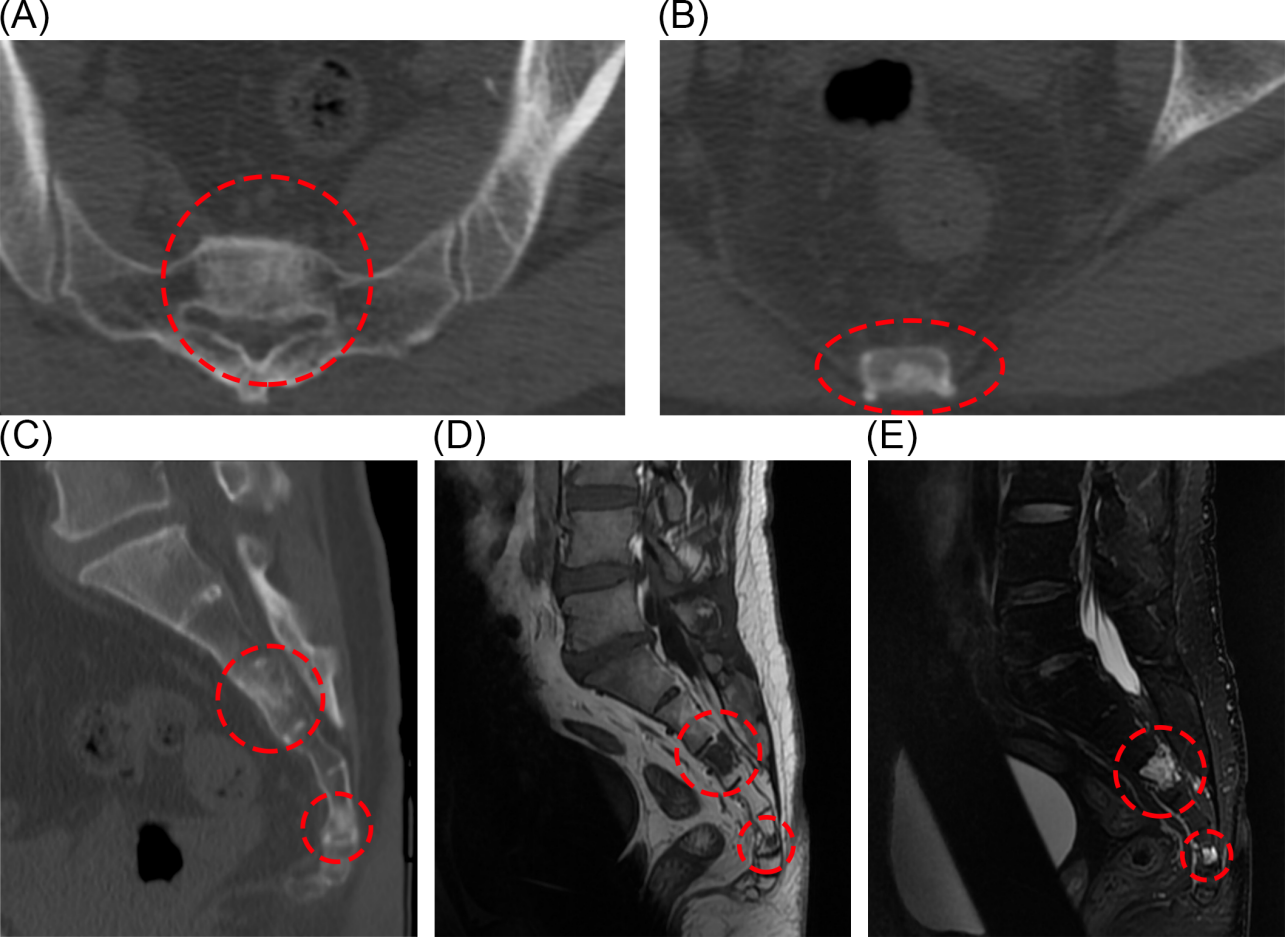
Postoperative CT showed abnormal signal foci in S3 and S5 cones **(A-C)**. Sacral lesions in the S3 and S5 pyramids can also be seen on MRI **(D, E)**.

After receiving the postoperative pathology report, we noted that BNCT typically occurs in intra-axial regions. Therefore, we recommended the patient undergo a spinal CT and a whole-body multimodal PET scan. CT images showed abnormal signal foci in the S3 and S5 vertebrae (**Figures 4A–C**), without bone destruction and osteolysis. In MRI images, lesions in S3 and S5 show low signal on T1 and high signal on T2 (**Figures 4D, E**). In the S3 and S5 sacral vertebrae, FDG metabolism was slightly increased in the lesions. These findings suggest the presence of lesions suspicious for benign tumors within the sacral spine. We will continue regular follow-up of the patients’ sacral lesions.

## 3 Discussion

More than 95% of solitary pulmonary nodules are benign lesions, such as intrapulmonary lymph nodes, granulomas, etc. Currently, differential diagnosis mainly relies on imaging features and pathological test results (2). The largest pulmonary nodule in this case is about 16mm×15mm. Previous case reports have documented similar pulmonary nodule sizes, with benign lesions including: pulmonary hamartoma (3), granulomatous infection (4) and sarcoidosis (5). Malignant lesions include lung adenocarcinoma (4), carcinoid tumor (6), prostate cancer lung metastasis (7) and liposarcoma (8).

The academic community’s understanding of BNCT has evolved significantly over the decades. The term was first introduced in 1952 by Congdon, who labeled BNCT as “Benign Chordomas” (9). Subsequently, various scholars have referred to these tumors using terms like “giant notochordal rest” (10) and “giant notochordal hamartoma” (11). Yamaguchi et al. (12) established the standard term “benign notochordal cell tumor” in 2004.

While most BNCTs are small and clinically insignificant, with over half of the lesions measuring less than 2 mm in diameter. There are rare instances of large tumors that compress surrounding tissues and cause symptoms (13). The tumor primarily appears at the base of the skull and spine, with autopsy reports indicating incidence rates of 11.5% in the clivus and 19% in the vertebrae (14). Notably, extra-axial BNCTs are exceedingly rare, with documented cases all occurring in the lungs (15–17). In their report, the diameters of the lung nodules that were ultimately diagnosed as BNCT were 15 mm, 12 mm, and 20 mm, respectively. None of them mentioned the presence of tumor lesions in the spine.

BNCT does not cause bone destruction and lacks soft tissue extension. On MRI, changes in signal intensity are evident. Histologically, BNCT predominantly consists of vacuolated cells resembling adipocytes, typically without an extracellular myxoid matrix and exhibiting only mild atypia. Immunohistochemically, BNCT expresses brachyury and keratins (CK8, CK18, CK19), and usually tests positive for epithelial membrane antigen (EMA), vimentin (Vim), and S-100 protein, which was consistent with this case (12, 18). Special attention should be paid to the differentiation of S-100-positive lesions with similar morphology, including lipoastrocytoma, liposarcoma, lipoma, adipocytes, chondroma, chondrosarcoma, glioma, and chordoma (19). Therefore, a comprehensive diagnostic approach including multiple imaging techniques and histopathological examination is particularly important for distinguishing BNCT.

The WHO classification of notochordal tumors includes Chordoma, BNCT, dedifferentiated chordoma, and poorly differentiated chordoma. The characteristics and pathological findings of the lung tumors in this case align closely with those of previously reported BNCTs. It is currently believed that primary BNCT in the lung results from notochord remnants that persist during embryonic development. Embryological studies suggest that notochord remnants can be present anywhere along the axial path of notochord evolution (20).

In most cases, BNCT is in a quiescent state and is generally considered to require no special treatment. Surgical resection is an effective method for removing lesions, and there are currently no reports of recurrence after BNCT surgery. But some scholars now suggest that BNCT may be a precursor lesion of chordoma (21). Unlike BNCT, chordoma is a highly malignant tumor characterized by intracytoplasmic mucus and eosinophils, and it tends to recur and metastasize, making it challenging to cure. Both BNCT and chordoma share similarities in immunophenotype (22, 23), ultrastructure (24, 25), and positive expression of Brachyury transcription factor (26), suggesting a common origin from the notochord during embryonic development. Furthermore, immunohistochemical studies have shown positive results for p-mTOR and p-p70 S6 in both chordoma and BNCT (27), indicating that the mTOR pathway, crucial for cell growth, proliferation, and metabolism, is active in these cells (28). Therefore, BNCT should be given enough attention and close follow-up.

The differential diagnosis between BNCT and chordoma is challenging. Compared with chordoma, BNCT has milder clinical manifestations, often without subjective symptoms, and rarely shows bone destruction in imaging. On pathological images, BNCT lacks cellular atypia, mitotic activity, vascularity, extracellular myxoid matrix, intracytoplasmic mucus, and eosinophils. Besides, researchers have utilized various genetic analysis methods to explore the pathogenesis of BNCT. For instance, Du et al. (18) examined 13 patients with skull base BNCT, revealing copy number aberrations in chromosomes 1, 3, 6, 7, 12, and 19 in all subjects. Unlike chordomas, chromosome gains or normal copy numbers are more common in BNCT, whereas chromosome losses are uncommon.

The management of BNCT mainly relies on imaging and histological features, which can be divided into typical BNCT and atypical/indeterminate lesions. For typical BNCT (imaging manifestations of lesions ≤35mm, no obvious clinical symptoms, no soft tissue extension, no bone destruction, and no contrast enhancement, Ki67 index ≤5%), regular imaging monitoring is recommended, and the first follow-up is a CT or MRI review at about 6 months. If there is no progression, the subsequent follow-up is once a year, and no surgical intervention is required. For atypical or indeterminate BNCT (such as imaging manifestations of enhanced signal, bone destruction, soft tissue extension, dura mater involvement, or lesion growth >1-2mm/year during follow-up, Ki67 index >5%), tissue biopsy is recommended to clarify the pathological nature. If the pathological examination indicates malignant transformation or a progressive trend, surgical resection should be considered, and radiotherapy or proton therapy can be combined after surgery to reduce the risk of recurrence. For patients with neurological symptoms, even if the imaging characteristics are consistent with typical BNCT, it is still necessary to evaluate whether to perform surgical intervention. All management decisions should be discussed by a multidisciplinary team (MDT) to optimize treatment strategies and avoid unnecessary treatment or delay in optimal intervention (29).

In this case, the sacral lesions appeared relatively regular on imaging, showed no signs of obvious invasive growth, and exhibited a slight increase in FDG metabolism. After discussion by our hospital’s MDT, it was considered that the lesions in the sacrum were benign and should be followed up regularly. If there is a tendency for malignancy, early intervention should be carried out. If necessary, we will perform surgical resection of the deteriorating sacral lesions with the patient’s consent, and we will send the pathological specimens to hospitals or institutions with more advanced equipment for histological and immunohistochemical testing. In addition to the pathological results mentioned in this article, we hope that future tests will include specific cytokeratin (such as CK8, CK18, and CK19) to supplement more pathological evidence. This case report highlights a rare instance of primary bilateral lung BNCTs concurrently presenting with sacral tumors, providing a new reference for thoracic surgeons in the diagnosis of pulmonary nodules. At the same time, we should fully consider the association between pulmonary nodules and diseases in other parts of the body.

## Data Availability

The original contributions presented in the study are included in the article/supplementary material. Further inquiries can be directed to the corresponding author.

## References

[1] Anderson WJ Doyle LA. Updates from the 2020 world health organization classification of soft tissue and bone tumours. Histopathology. (2021) 78:644–57. doi: 10.1111/his.14265 33438273

[2] Mazzone PJ Lam L. Evaluating the patient with a pulmonary nodule: A review. JAMA. (2022) 327:264–73. doi: 10.1001/jama.2021.24287 35040882

[3] Itoga M Kobayashi Y Takeda M Moritoki Y Tamaki M Nakazawa K. A case of pulmonary hamartoma showing rapid growth. Case Rep Med. (2013) 2013:231652. doi: 10.1155/2013/231652 24171003 PMC3792529

[4] Watanabe H Uruma T Seita I Oishi T Watanabe Y Tsukimori A. Solitary pulmonary caseating granulomas: A 5-year retrospective single-center analysis. Mol Clin Oncol. (2017) 6:839–45. doi: 10.3892/mco.2017.1244 PMC5451854 28588774

[5] Razdan PS Buteau D Pollock NW. A case of Löfgren’s syndrome confused with decompression sickness. Diving Hyperb Med. (2019) 49:306–10. doi: 10.28920/dhm49.4.306-310 PMC7039773 31828751

[6] Steinfort DP Finlay M Irving LB. Diagnosis of peripheral pulmonary carcinoid tumor using endobronchial ultrasound. Ann Thoracic Med. (2008) 3:146. doi: 10.4103/1817-1737.43082 PMC2700446 19561897

[7] Tarabaih M Degheili JA Nasser M. Isolated solitary lung nodule in a patient with idiopathic pulmonary fibrosis and concomitant prostate cancer: A challenging diagnosis. Cureus. (2021) 13:e14218. doi: 10.7759/cureus.14218 33948408 PMC8086736

[8] Yamamoto J Yamauchi Y Nakano R Shiraiwa Y Ikeda T Kusumoto T. Well differentiated liposarcoma of a lung: report of a case. Kyobu Geka. (2024) 77:230–4. 38465498

[9] Congdon CC. Benign and malignant chordomas: a clinico-anatomical study of twenty-two cases. Am J Pathol. (1952) 28:793–821. 12976524 PMC1937384

[10] Kyriakos M Totty WG Lenke LG. Giant vertebral notochordal rest: A lesion distinct from chordoma: discussion of an evolving concept. Am J Surg Pathol. (2003) 27:396. doi: 10.1097/00000478-200303000-00015 12604898

[11] Mirra JM Brien EW. Giant notochordal hamartoma of intraosseous origin: a newly reported benign entity to be distinguished from chordoma. Report of two cases. Skeletal Radiol. (2001) 30:698–709. doi: 10.1007/s002560100422 11810168

[12] Yamaguchi T Suzuki S Ishiiwa H Shimizu K Ueda Y. Benign notochordal cell tumors: A comparative histological study of benign notochordal cell tumors, classic chordomas, and notochordal vestiges of fetal intervertebral discs. Am J Surg Pathol. (2004) 28:756. doi: 10.1097/01.pas.0000126058.18669.5d 15166667

[13] Krisht KM Palmer CA Osborn AG Couldwell WT. Giant ecchordosis physaliphora in an adolescent girl. J Neurosurg. (2013) 12(4):328–33. doi: 10.3171/2013.5.PEDS1395 23909619

[14] Bjornsson J Wold LE Ebersold MJ Laws ER. Chordoma of the mobile spine. A clinicopathologic analysis of 40 patients. Cancer. (1993) 71:735–40. doi: 10.1002/1097-0142(19930201)71:3<735::AID-CNCR2820710314>3.0.CO;2-8 8431853

[15] Kikuchi Y Yamaguchi T Kishi H Azuhata K Kimizuka G Hiroshima K. Pulmonary tumor with notochordal differentiation: report of 2 cases suggestive of benign notochordal cell tumor of extraosseous origin. Am J Surg Pathol. (2011) 35:1158. doi: 10.1097/PAS.0b013e318220e085 21716081

[16] Shintaku M Kikuchi R. Benign notochordal cell tumor of the lung: Report of a case. Pathol Int. (2020) 70:871–5. doi: 10.1111/pin.13005 32827236

[17] Strano S Ouafi L Baud M Alifano M. Primary chordoma of the lung. Ann Thorac Surg. (2010) 89:302–3. doi: 10.1016/j.athoracsur.2009.05.043 20103267

[18] Du J Xu L Cui Y Liu Z Su Y Li G. Benign notochordal cell tumour: clinicopathology and molecular profiling of 13 cases. J Clin Pathol. (2019) 72:66–74. doi: 10.1136/jclinpath-2018-205441 30355586

[19] Grabovska D Strumfa I Ositis J Liepniece-Karele I Balodis A. Benign notochordal cell tumours: case report and literature review. Diagnostics. (2024) 14:1330. doi: 10.3390/diagnostics14131330 39001221 PMC11240483

[20] Willis RA. THE BORDERLAND OF EMBRYOLOGY AND PATHOLOGY (ed. 2). Obstetrics Gynecol. (1963) 22:695.

[21] Kreshak J Larousserie F Picci P Boriani S Mirra J Merlino B. Difficulty distinguishing benign notochordal cell tumor from chordoma further suggests a link between them. Cancer Imaging. (2014) 14:4. doi: 10.1186/1470-7330-14-4 25609192 PMC4212531

[22] Amer HZM Hameed M. Intraosseous benign notochordal cell tumor. Arch Pathol Lab Med. (2010) 134:283–8. doi: 10.5858/134.2.283 20121620

[23] Macdonald RL Deck JHN. Immunohistochemistry of ecchordosis physaliphora and chordoma. Can J Neurological Sci. (1990) 17:420–3. doi: 10.1017/S0317167100031000 1703453

[24] Ho KL. Ecchordosis physaliphora and chordoma: a comparative ultrastructural study. Clin Neuropathol. (1985) 4:77–86. 3995810

[25] Kyriakos M. Benign notochordal lesions of the axial skeleton: a review and current appraisal. Skeletal Radiol. (2011) 40:1141–52. doi: 10.1007/s00256-011-1167-6 21847746

[26] Vujovic S Henderson S Presneau N Odell E Jacques T Tirabosco R. Brachyury, a crucial regulator of notochordal development, is a novel biomarker for chordomas. J Pathol. (2006) 209:157–65. doi: 10.1002/path.1969 16538613

[27] Presneau N Shalaby A Idowu B Gikas P Cannon SR Gout I. Potential therapeutic targets for chordoma: PI3K/AKT/TSC1/TSC2/mTOR pathway. Br J Cancer. (2009) 100:1406–14. doi: 10.1038/sj.bjc.6605019 PMC2694420 19401700

[28] Dobashi Y Suzuki S Sato E Hamada Y Yanagawa T Ooi A. EGFR-dependent and independent activation of Akt/mTOR cascade in bone and soft tissue tumors. Modern Pathol. (2009) 22:1328–40. doi: 10.1038/modpathol.2009.104 19648884

[29] Usher I Flanagan AM Choi D. Systematic review of clinical, radiologic, and histologic features of benign notochordal cell tumors: implications for patient management. World Neurosurgery. (2019) 130:13–23. doi: 10.1016/j.wneu.2019.06.009 31421435

